# Supplementary material for: Cross-Talk between N6-Methyladenosine and Their Related RNAs Defined a Signature and Confirmed m6A Regulators for Diagnosis of Endometriosis
Source: Int J Mol Sci. 2023 Jan 14;24(2):1665. doi: 10.3390/ijms24021665 (PMC9862014; doi:10.3390/ijms24021665)
Supplement: Supplementary file 1 [file ijms-24-01665-s001.zip › ijms-2134749-supplementary.pdf]

International Journal of
Molecular Sciences

MDPI

Supplementary Materials

Table S1. Primer sequences Table.

Gene Name	Primers Sequences
YTHDF2	Forward:GGTTCTGTGCATCAAAAGGATGG<br>Reverse:CCAAAGAATAGGAAAAGCCAATGG
METTL3	Forward:ACCCTGACAGATGATGAGATGC<br>Reverse:CGTTCATACCCCCAGAGGTTTAG
FTO	Forward:TGGTGTCCCAAGAAATCGTG<br>Reverse:TGCAGGCCGTGAACCAC
GAPDH	Forward:GGAGCGAGATCCCTCCAAAAT<br>Reverse:GGCTGTTGTCATACTTCTCATGG

Table S2. The entire results of GSEA.

Name	GS<br>follow Link to MSigDB	Size	ES	NES	NOM *p*-val	FDR q-val	FWER *p*-val	Rank at Max	Leading Edge
HALLMARK_TNFA_SIGNALING_VIA_NFKB	HALLMARK_TNFA_SIGNALING_VIA_NFKB	161	−0.64128256	−3.4475446	0	0	0	2487	tags = 71%, list = 25%, signal = 94%
HALLMARK_INFLAMMATORY_RESPONSE	HALLMARK_INFLAMMATORY_RESPONSE	142	−0.61479205	−3.2378798	0	0	0	2487	tags = 67%, list = 25%, signal = 88%
HALLMARK_IL6_JAK_STAT3_SIGNALING	HALLMARK_IL6_JAK_STAT3_SIGNALING	62	−0.62766963	−2.8894393	0	0	0	1654	tags = 60%, list = 17%, signal = 71%
HALLMARK_COAGULATION	HALLMARK_COAGULATION	91	−0.56792736	−2.8482537	0	0	0	2536	tags = 62%, list = 25%, signal = 82%
HALLMARK_ALLOGRAFT_REJECTION	HALLMARK_ALLOGRAFT_REJECTION	131	−0.5079948	−2.6696498	0	0	0	2076	tags = 55%, list = 21%, signal = 68%
HALLMARK_HYPOXIA	HALLMARK_HYPOXIA	155	−0.48197454	−2.6499376	0	0	0	2512	tags = 51%, list = 25%, signal = 67%
HALLMARK_INTERFERON_GAMMA_RESPONSE	HALLMARK_INTERFERON_GAMMA_RESPONSE	157	−0.47960755	−2.6322768	0	0	0	2385	tags = 49%, list = 24%, signal = 63%
HALLMARK_EPITHELIAL_MESENCHYMAL_TRANSITION	HALLMARK_EPITHELIAL_MESENCHYMAL_TRANSITION	170	−0.46156034	−2.5528734	0	0	0	2920	tags = 55%, list = 29%, signal = 76%

HALLMARK_COMPLEMENT	HALLMARK_COMPLEMENT	143	−0.47518152	−2.491453	0	0	0	1697	tags = 41%, list = 17%, signal = 49%
HALLMARK_KRAS_SIGNALING_UP	HALLMARK_KRAS_SIGNALING_UP	142	−0.43182883	−2.2495372	0	0	0	1952	tags = 39%, list = 20%, signal = 48%
HALLMARK_APOPTOSIS	HALLMARK_APOPTOSIS	112	−0.42601973	−2.1714253	0	0	0	1517	tags = 37%, list = 15%, signal = 43%
HALLMARK_P53_PATHWAY	HALLMARK_P53_PATHWAY	147	−0.4030426	−2.137378	0	0	0	2277	tags = 46%, list = 23%, signal = 59%
HALLMARK_MYOGENESIS	HALLMARK_MYOGENESIS	129	−0.40888703	−2.1307166	0	0	0	2724	tags = 47%, list = 27%, signal = 63%
HALLMARK_IL2_STAT5_SIGNALING	HALLMARK_IL2_STAT5_SIGNALING	151	−0.39225355	−2.096334	0	0	0	1849	tags = 35%, list = 18%, signal = 42%
HALLMARK_APICAL_JUNCTION	HALLMARK_APICAL_JUNCTION	127	−0.37918523	−2.0371587	0	2.67E−04	0.001	2646	tags = 46%, list = 26%, signal = 61%
HALLMARK_REACTIVE_OXYGEN_SPECIES_PATHWAY	HALLMARK_REACTIVE_OXYGEN_SPECIES_PATHWAY	38	−0.46128616	−1.9620595	0	5.67E−04	0.003	2011	tags = 47%, list = 20%, signal = 59%
HALLMARK_INTERFERON_ALPHA_RESPONSE	HALLMARK_INTERFERON_ALPHA_RESPONSE	80	−0.389426	−1.8728471	0	9.52E−04	0.005	2878	tags = 49%, list = 29%, signal = 68%
HALLMARK_TGF_BETA_SIGNALING	HALLMARK_TGF_BETA_SIGNALING	42	−0.44365442	−1.8168998	0.003703704	0.001676679	0.01	1018	tags = 33%, list = 10%, signal = 37%
HALLMARK_APICAL_SURFACE	HALLMARK_APICAL_SURFACE	26	−0.4834605	−1.7981799	0	0.001588433	0.01	3823	tags = 69%, list = 38%, signal = 112%
HALLMARK_UV_RESPONSE_UP	HALLMARK_UV_RESPONSE_UP	113	−0.30922306	−1.582043	0	0.013841847	0.075	2030	tags = 38%, list = 20%, signal = 47%
HALLMARK_CHOLESTEROL_HOMEOSTASIS	HALLMARK_CHOLESTEROL_HOMEOSTASIS	54	−0.28831092	−1.3189623	0.04109589	0.08499916	0.421	1015	tags = 20%, list = 10%,

									signal = 23%
HALLMARK_PI3K_AKT_MTOR_SIGNALING	HALLMARK_PI3K_AKT_MTOR_SIGNALING	66	−0.2823685	−1.3072615	0.07655502	0.088084966	0.45	1449	tags = 29%, list = 14%, signal = 33%
HALLMARK_XENOBIOTIC_METABOLISM	HALLMARK_XENOBIOTIC_METABOLISM	147	−0.23496242	−1.2646426	0.019607844	0.11313333	0.554	1815	tags = 24%, list = 18%, signal = 29%
HALLMARK_HEME_METABOLISM	HALLMARK_HEME_METABOLISM	129	−0.23603305	−1.2606056	0.048484847	0.111276954	0.561	2459	tags = 30%, list = 25%, signal = 40%
HALLMARK_ANGIOGENESIS	HALLMARK_ANGIOGENESIS	29	−0.3294821	−1.2601901	0.124590166	0.107048914	0.562	2478	tags = 48%, list = 25%, signal = 64%
HALLMARK_ESTROGEN_RESPONSE_EARLY	HALLMARK_ESTROGEN_RESPONSE_EARLY	148	−0.23081213	−1.2263274	0.06923077	0.13285577	0.655	2896	tags = 36%, list = 29%, signal = 50%
HALLMARK_KRAS_SIGNALING_DN	HALLMARK_KRAS_SIGNALING_DN	84	−0.24127336	−1.1828799	0.165	0.17149375	0.751	2993	tags = 38%, list = 30%, signal = 54%
HALLMARK_NOTCH_SIGNALING	HALLMARK_NOTCH_SIGNALING	23	−0.2753826	−0.9841351	0.48765433	0.5623192	0.993	3319	tags = 48%, list = 33%, signal = 71%
HALLMARK_GLYCOLYSIS	HALLMARK_GLYCOLYSIS	157	−0.168284	−0.9275799	0.75	0.7010433	0.997	1602	tags = 20%, list = 16%, signal = 23%
HALLMARK_UV_RESPONSE_DN	HALLMARK_UV_RESPONSE_DN	102	−0.16506612	−0.8237863	0.90697676	0.91158783	0.999	1516	tags = 19%, list = 15%, signal = 22%
HALLMARK_ANDROGEN_RESPONSE	HALLMARK_ANDROGEN_RESPONSE	82	−0.1543443	−0.76540107	0.92783505	0.952295	0.999	2340	tags = 26%, list = 23%, signal = 33%
HALLMARK_ADIPOGENESIS	HALLMARK_ADIPOGENESIS	165	−0.13757534	−0.755156	0.98373985	0.9320165	0.999	3083	tags = 35%, list = 31%, signal = 49%

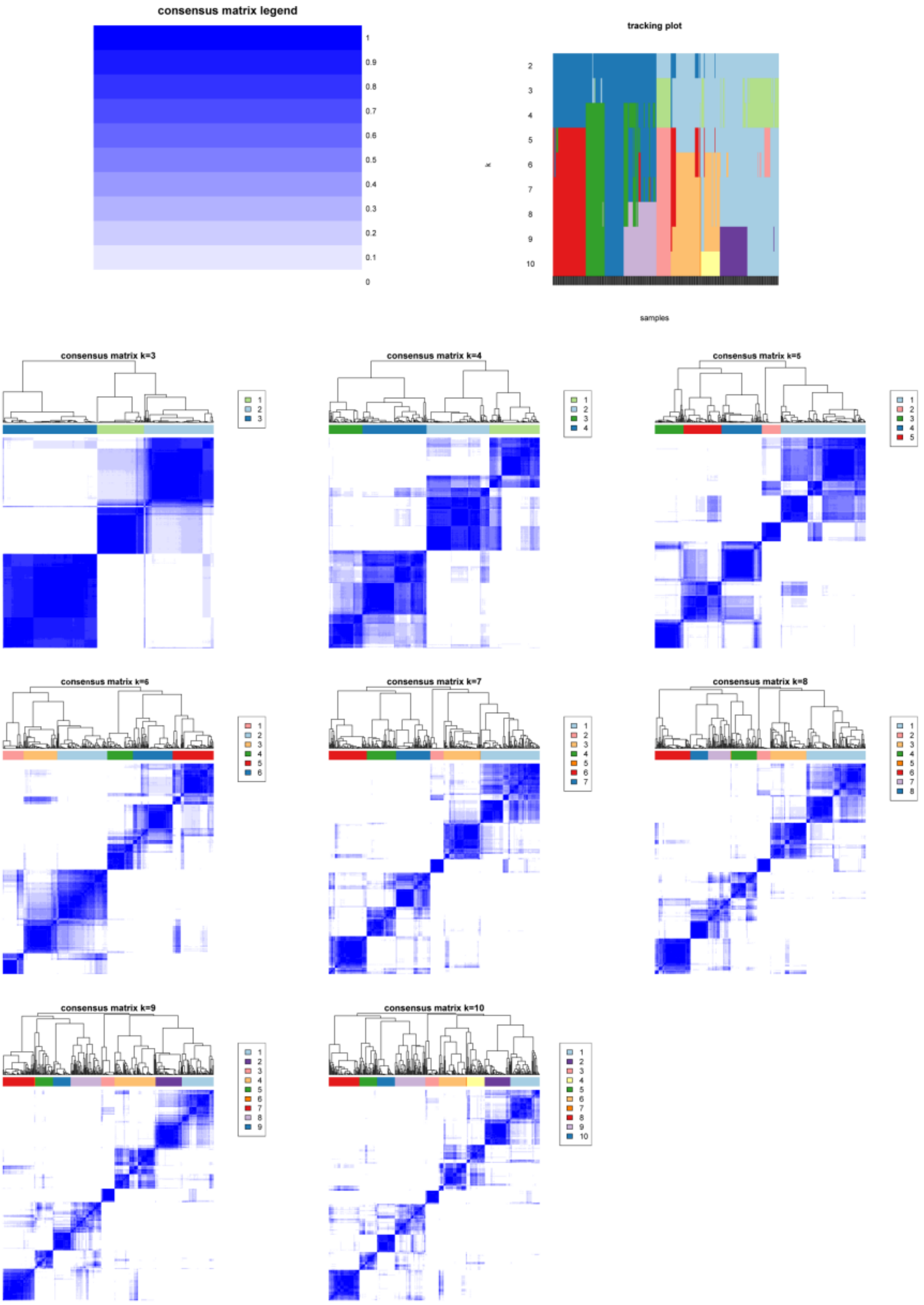

Figure S1. Consensus clustering analysis showing additional consensus matrix and tracking plot from k = 3 to k =10.

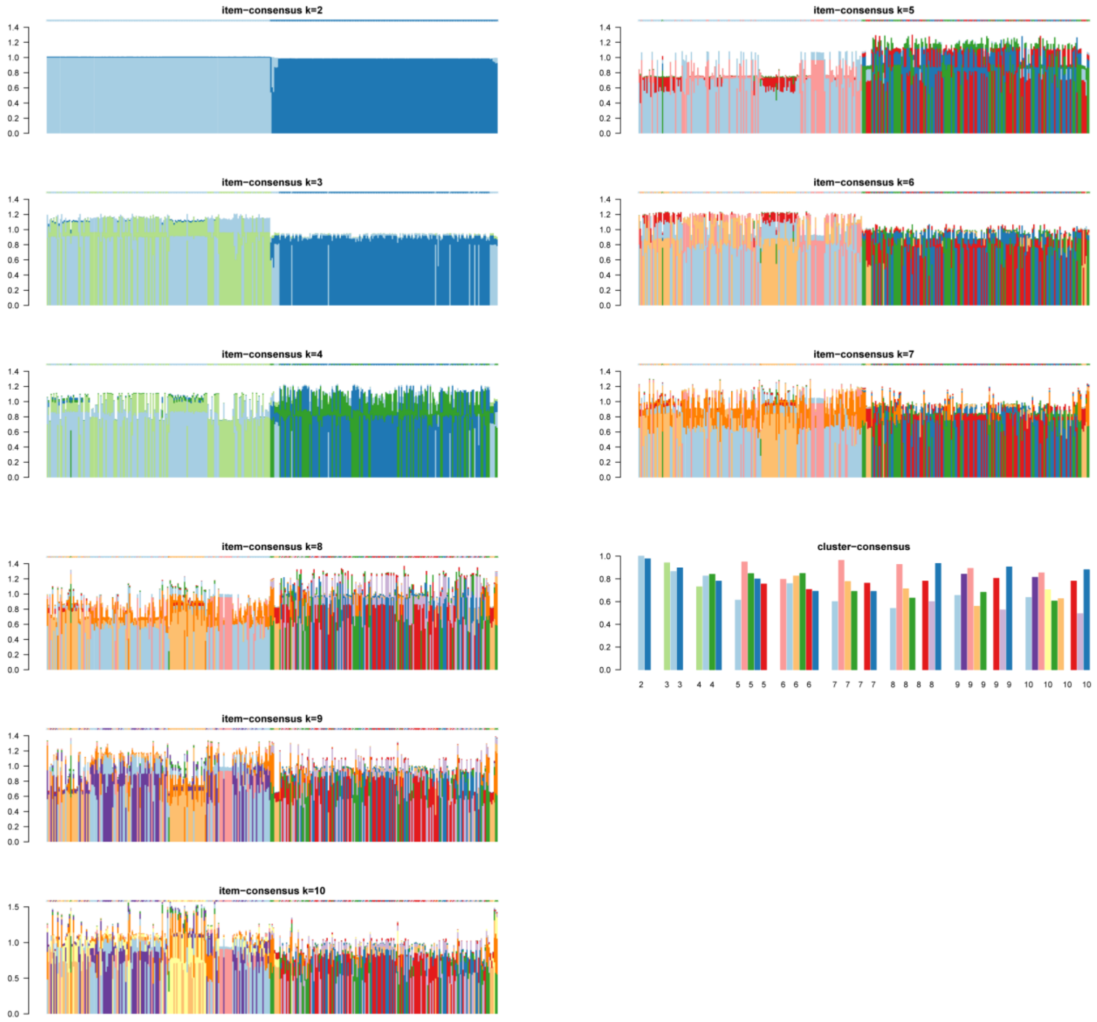

Figure S2. Consensus clustering analysis showing all consensus k values.

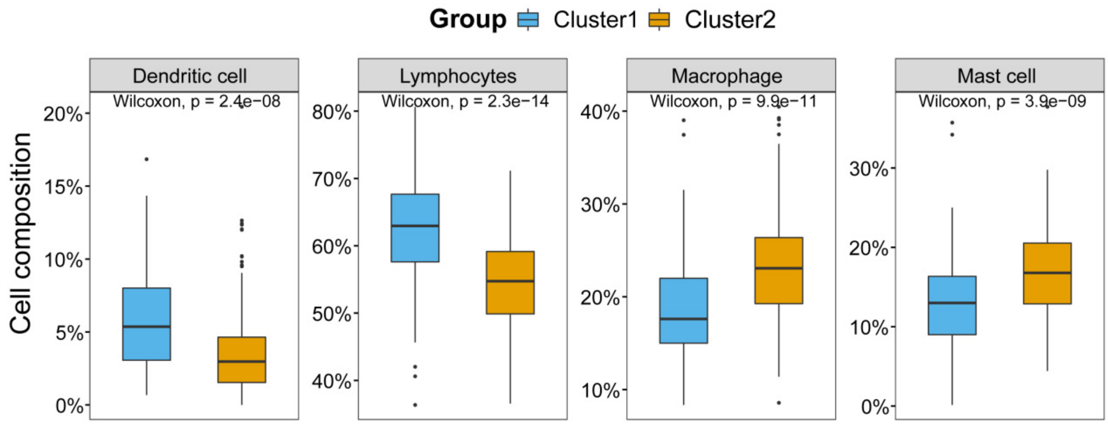

Figure S3. The composition of total lymphocytes, total dendritic cells, total macrophages, and total mast cells between two consensus subtypes tested by Wilcoxon rank sum analysis.

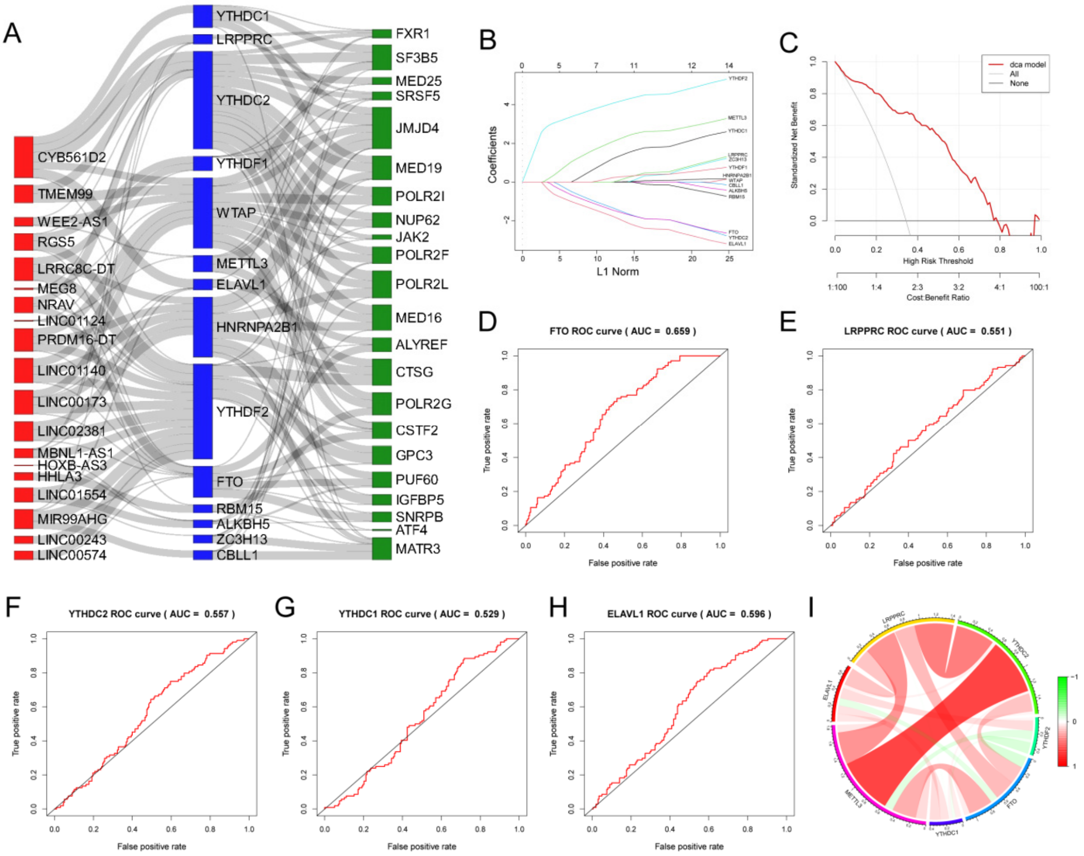

Figure S4. (**A**) The Sankey plot showed interactions among 14 key m6A regulators (middle column) and lncRNAs (left column) and mRNAs (right column). Width of each alluvial stripe represents pairwise Pearson's correlation. (**B**) Extracting parameters using LASSO. (**C**) The DCA plot of m6A diagnostic model in GSE141549. The roc plots for FTO (**D**), LRPPRC (**E**), YTHDC2 (**F**), YTHDC1 (**G**), and ELAVL1 (**H**). (**I**) The mutual relationship among the composition of the diagnostic model in circos plot in eutopic samples. LASSO, least absolute shrinkage and selection operator; ROC, receiver operating characteristic; AUC, area under curve.
